# Social transmission of bacterial symbionts homogenizes the microbiome within and across generations of group-living spiders

**DOI:** 10.1038/s43705-023-00256-2

**Published:** 2023-06-17

**Authors:** Clémence Rose, Marie B. Lund, Andrea M. Søgård, Mette M. Busck, Jesper S. Bechsgaard, Andreas Schramm, Trine Bilde

**Affiliations:** 1 Section for Genetics, Ecology and Evolution, Department of Biology, Aarhus University, Aarhus, Denmark; 2 Section for Microbiology, Department of Biology, Aarhus University, Aarhus, Denmark

**Keywords:** Microbial ecology, Symbiosis

## Abstract

Disentangling modes and fidelity of symbiont transmission are key for understanding host–symbiont associations in wild populations. In group-living animals, social transmission may evolve to ensure high-fidelity transmission of symbionts, since non-reproducing helpers constitute a dead-end for vertical transmission. We investigated symbiont transmission in the social spider *Stegodyphus dumicola*, which lives in family groups where the majority of females are non-reproducing helpers, females feed offspring by regurgitation, and individuals feed communally on insect prey. Group members share temporally stable microbiomes across generations, while distinct variation in microbiome composition exists between groups. We hypothesized that horizontal transmission of symbionts is enhanced by social interactions, and investigated transmission routes within (horizontal) and across (vertical) generations using bacterial 16S rRNA gene amplicon sequencing in three experiments: (i) individuals were sampled at all life stages to assess at which life stage the microbiome is acquired. (ii) a cross-fostering design was employed to test whether offspring carry the microbiome from their natal nest, or acquire the microbiome of the foster nest via social transmission. (iii) adult spiders with different microbiome compositions were mixed to assess whether social transmission homogenizes microbiome composition among group members. We demonstrate that offspring hatch symbiont-free, and bacterial symbionts are transmitted vertically across generations by social interactions with the onset of regurgitation feeding by (foster)mothers in an early life stage. Social transmission governs horizontal inter-individual mixing and homogenization of microbiome composition among nest mates. We conclude that temporally stable host–symbiont associations in social species can be facilitated and maintained by high-fidelity social transmission.

## Introduction

Host–symbiont relationships range from tight associations, where the partners cannot survive without each other, to highly flexible and facultative associations that can be entirely environmentally determined [1–3]. It is becoming increasingly clear that host-associated microbiomes can differ widely among individuals and populations of the same species in affinity, species composition, and abundance across different temporal and spatial scales [4–9]. To understand the temporal and spatial variation in microbiome composition and dynamics observed in wild populations, it is key to establish how the microbiome is transmitted within and across generations [10, 11]. Historically, symbiont transmission has been studied under two different strategies: vertical transmission takes place when symbionts are transferred from the parental generation to the offspring, i.e., across generations, whereas horizontal transmission happens when symbionts are acquired from the environment or from co-occurring host individuals within the same generation. Vertical transmission can occur through the germ line from mother to offspring, or by other modes of transmission from the parental generation to the offspring. Vertical and horizontal transmission modes are not mutually exclusive, and symbiont transmission can be facilitated by a combination of both modes of transmission, i.e., mixed mode transmission [12]. Both facultative and obligate associations can be maintained by vertical or horizontal transmission, or by a combination of both, with important consequences for shaping host–symbiont relationships, e.g., partner fidelity and symbiont genome evolution [10, 12–14]. Although theory predicts that stable associations are best governed by vertical transmission, there are many examples of horizontal transmission governing tight host–symbiont relationships. The establishment of stable symbioses may further rely on a critical temporal acquisition window: in corals, the acquisition of photosymbionts at the juvenile stage is not beneficial for the host [15]; the infection of the stinkbug *Riptortus pedetris* by its beneficial symbiont Burkholderia is determined by host development and life stage [16]; the nephridial symbionts of earthworms only colonize the embryo before the opening of the nephridial pore [17], and transmission of *Actinobacteria* symbionts occurs at a very narrow post-eclosion inoculation period in in leaf-cutting ants [18]. The complexity of these examples highlights the importance not only of disentangling vertical and horizontal modes of transmission and their fidelity, but also the timing in the development of the host for when symbiont infections occur, to shed light on the formation of stable host–symbiont associations.

Social interactions can mediate partial or complete symbiont transmission among host individuals [19–22]. Group living enhances the likelihood of social transmission of symbionts because of the close proximity and frequent interactions among host individuals [20, 21]. Social interactions such as grooming, or mouth-to-mouth feeding, should promote repeated inoculations governing transmission, exchange, and homogenization of symbionts among individuals within and between generations [21–24]. A close association between social transmission and group living is found in different taxa, with examples including primates [24], mice [23], honey bees [25], bumble bees [26], and termites [27]. Social transmission has the capacity to maintain host–symbiont associations of both intracellular (e.g., *Wolbachia* in ants [28] and *Arsenophonus* in honey bees [29]) and extracellular symbionts (e.g., *Streptomyces* in ants [30]), gut microbiota in social bees, reviewed in [31]) and can be an important driver for the evolution of symbiosis and group living [19, 20].

In spiders, sociality has evolved independently in at least 22 species within 6 families [32]. The social species build communal nests and capture webs with hundreds of inbred individuals, which cooperate on prey capture and offspring care [32]. Microbiomes have been studied for the three social species of the genus *Stegodyphus* (Eresidae), *S. sarasinorum*, *S. mimosarum*, and in particular for *S. dumicola* [33, 34]. This species occurs in southern Africa and harbors a low-diversity bacterial microbiome with five core endosymbionts (prevalence >50%), of which one or two typically dominate an individual spider [33]. The endosymbionts include spider-specific relatives of the genus *Mycoplasma* and *Borellia* (*Ca*. Arachnospira), and the obligate intracellular arthropod symbiont *Diplorickettsia*; none of them appears to be obligate for the spider host. Within a social group (nest), microbiome composition is very similar among individuals and over time, both within and across generations, but it can vary significantly among social groups within a local area [4, 33]. While it is unknown to what extent social interactions shape this pattern [33, 35, 36], several characteristics of the social spider biology would facilitate social transmission: first, living in communal nests entails a high frequency of social interactions. Second, communal feeding leads to an exchange of digestive fluids and possibly endosymbionts, when several spiders inject digestive fluids into the same prey and suck up a mixture of these fluids and digested prey [32, 37]. Third, only approximately one-third of the females reproduce, but all of them provide extended maternal care by regurgitation feeding of the offspring with a mixture of digested prey and dissolved intestinal lining [38]. This is followed by the consumption of all females (matriphagy) once the offspring are ready to catch prey independently [32, 39]. The combination of reproductive skew and allomaternal care by helper females is predicted to favor horizontal transmission, since non-reproductive individuals would represent a dead-end for vertically transmitted symbionts [28, 40]. On the other hand, regurgitation feeding and matriphagy also provide a route for vertical transmission across generations from the mother or from the helping females (allomothers) to offspring.

We, therefore, hypothesized that (i) endosymbionts in *S. dumicola* are maintained by horizontal transmission, at least partially mediated by social interactions. (ii) Social transmission occurs among group members and acts to homogenize the microbiome within a nest. We tested these hypotheses by a combination of life cycle analyses, cross-fostering experiments, and mixing of adult spiders with different endosymbionts to trace transmission routes within and across generations using 16S rRNA gene amplicon sequencing and quantitative polymerase chain reaction (qPCR).

## Material and methods

### Spider collection and maintenance

Nests containing family groups of spiders were collected in Namibia and Botswana between April 2017 and November 2019 (Supplementary Table S1) and transported to the lab in Aarhus University, Denmark. Spiders were kept in a 13:11 light cycle with a fluctuating temperature from 20° (night) to 29 °C (day); (7.30–8.30 a.m.: from 20° to >24°; 8.30 a.m.–8.30 p.m.: from 24° to >29°; 8.30–9.30 p.m.: from 29° to >24°; 9.30 pm: from 24° to >20°). Spiders were watered every day and fed twice a week with crickets (*Gryllus bimaculatus*) and blue bottle flies (*Calliphora vomitoria*). Based on previous studies, we know that the microbiomes of individual spiders are highly similar within a nest [4, 33]. Thus, in the following experiments, 2–3 spiders were considered to be representative of the individual microbiomes of the entire nest and/or life stages.

### Life cycle experiment

**Fig. 1:**
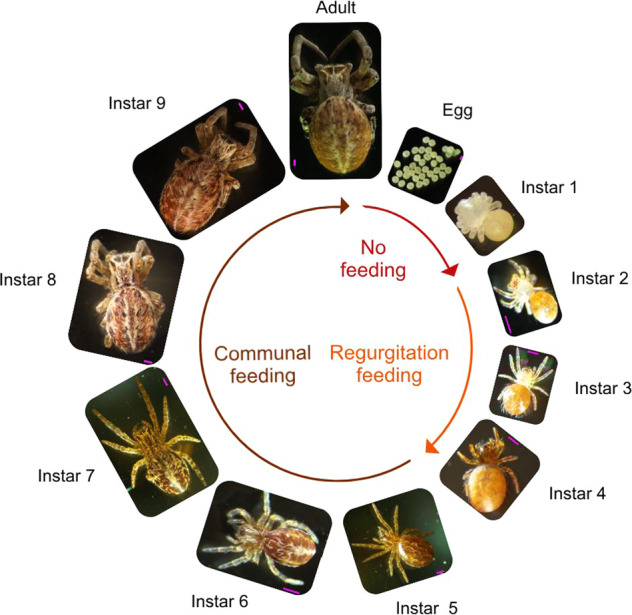
Life stages of the social spider *Stegodyphus dumicola* that were sampled during different stages of development. Eggs and Instar 1 occur inside egg sacs. Matriphagy (the consumption of mothers and female helpers, by large juveniles) occurs in instars 5–7. Pink bars are 500 µm.

**Fig. 2:**
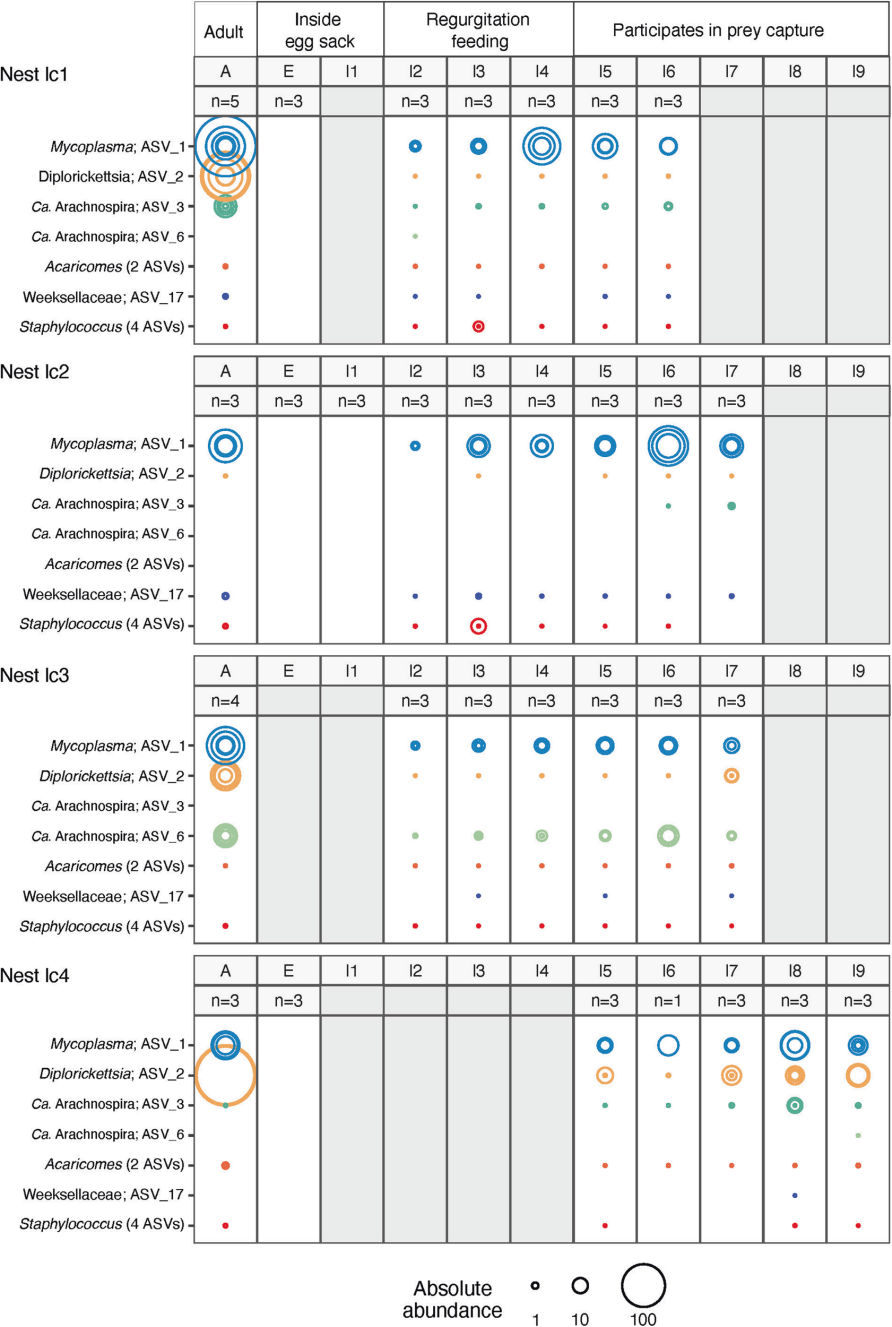
Absolute abundances in 16S rRNA gene copies per host gene of the main ASVs (bacterial symbionts) at different life stages in the social spider *S. dumicola* (A= Adult, E = Egg, I1-9= Instar one to nine) in four different nests. Sample size is indicated for each life stage. Life stages that could not be sampled are colored in gray and left empty. The size of the circle indicates the absolute abundance of the ASV. Colors correspond to symbiont taxonomy.

We investigated bacterial symbiont transmission across the life cycle of *S. dumicola*, to determine whether females deposit bacterial symbionts in the eggs, or whether and at which stage of the life cycle the offspring acquire microbial symbionts. Females produce egg sacs of spider silk which encapsulate the eggs. First instar offspring hatch inside the egg sac and after one molt, second instar offspring emerge from the egg sac. The different instars can be recognized by morphological characteristics such as size, presence of hairs, and by feeding behavior (Fig. 1). We monitored four nests with adult females that harbored different bacterial symbiont communities based on 16S rRNA gene amplicon sequencing (see “Adult” columns in Fig. 2 and below for method details), and for each developmental instar from egg to adult, we sampled three individuals from each nest for analyses. Samples were collected for DNA extraction, 16S rRNA gene amplicon sequencing, and quantitative PCR. Spiders were collected in 1.5 mL microcentrifuge tubes and immediately placed at −80 °C until further processing.

### Cross-fostering experiment

**Fig. 3:**
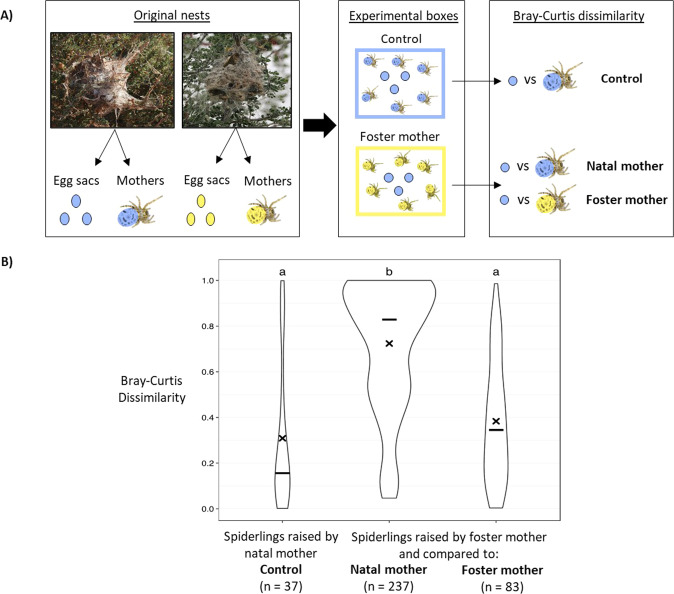
Cross-fostering experiment. **A** Experimental scheme. Females received either eggsac coming from their original nest (control, natal mother, blue) or from another nest (foster mother, yellow). Microbiome composition of the offspring raised by their natal mother was compared to that of their natal mother (control) while the microbiome composition of offspring raised by foster mother was compared to that of their natal mother (Natal mother) or their foster mother (Foster mother). **B** Bray-Curtis dissimilarity between the microbiomes of offspring and that of their natal mothers or foster mothers. Letters indicate significant differences between the two groups (Wilcoxon rank sum test, FDR-adjusted *p* value < 0.001).

The experiment was carried out in two rounds; the first experiment was conducted in winter (northern hemisphere) 2019 with six nests collected in October 2018 in Botswana. The second experiment was conducted in the spring (northern hemisphere) 2020 with seven nests collected in November 2019 in Namibia (Table S1). For both experiments, the microbiome composition of 2 individual adult female spiders from each nest was analyzed by 16S rRNA gene amplicon sequencing. For the cross-fostering experiments, we selected egg sacs originating from nests that differed in microbiome composition from that of foster females (Table 1). Thirteen nests were used for the cross-fostering experiment, two of which were used only for the control/natal nest treatment, while 11 nests were used for both control and cross-fostering treatments. Females were randomly chosen from each nest and placed in experimental boxes, each containing 15 adult females. Egg sacs were collected from the nests and marked with non-toxic acrylic paint to keep track of nest origin, and then distributed among the experimental boxes. Thirteen experimental boxes received an egg sac from the same nest as the females originated from (control, natal nest) and 15 experimental boxes received an egg sac from a nest with a different microbiome (foster nest) (Table 1; Fig. 3A). Boxes were checked every day to remove unmarked newly produced egg sacs. After hatching, the offspring were left to grow until they started to participate in prey capture and feed by themselves (instar 5, Fig. 1). Three individuals of each instar 5–9 and one female per experimental box were sampled in a 1.5 mL microcentrifuge tube and immediately stored at −80 °C for subsequent DNA extraction and 16S rRNA gene amplicon sequencing. Microbiome composition of the offspring raised by their natal mother was compared to that of their natal mother (control), and microbiome composition of offspring raised by a foster mother was compared to that of their natal mother or their foster mother, using a Wilcoxon rank sum test with FDR-adjusted *p*-values.

**Table 1:** Dominance and Shannon Index and relative abundances of the most abundant ASVs^a^ in nests used for the cross-fostering or mixed microbe experiments.

Nest	Dominance Index	Shannon Index	*Mycoplasma*			*Ca*. Arachno-spira	*Diplo-rickettsia*	*Acaricomes*	Experiment	Received egg sacs from
			ASV_1	ASV_4	ASV_5	ASV_3	ASV_2	ASV_8		
**es1**	0.993	0.066	0	0	0	0.99	0	0	Cross fostering	es1, es2, es4, es6
**es2**	0.762	1.310	0	0	0.57	0.15	0	0	Cross fostering	es2, es3, es6
**es3**	0.999	0.101	0	0	0	0.02	0.98	0	Cross fostering	es3, es6
**es4**	0.645	1.874	0	0	0	0	0	0.58	Cross fostering	es4
**es5**	0.997	0.076	0	0	0.99	0.01	0	0	Cross fostering	es3, es5
**es6**	0.995	0.724	0.53	0	0	0.46	0	0	Cross fostering	es3, es6
**es10**	0.925	0.765	0.06	0	0.18	0.02	0.75	0	Cross fostering	es10, es11
**es11**	0.998	0.080	0.99	0	0	0.01	0	0	Cross fostering	es10, es11
**es12**	1.000	0.014	0	0	0	0.99	0	0	Cross-fostering, Mixed microbe	es12, es15
**es13**	0.977	0.412	0.91	0.05	0	0	0	0.02	Cross fostering	es13, es14
**es14**	1.000	0.012	1	0	0	0	0	0	Cross fostering	es13, es14
**es15**	0.994	0.097	0.01	0.98	0	0	0	0.01	Cross-fostering, Mixed microbe	es15
**es16**	0.977	0.653	0	0	0.24	0.02	0.74	0	Cross fostering	es10, es11, es16
**mm1**	0.943	0.589	0.02	0	0.11	0.03	0.83	0	Mixed microbe	

^a^We calculated the mean relative abundance of the ASVs across all samples. We selected the most abundant ASVs present in the nests used both for the cross-fostering and mixed microbe experiments, as we know these six ASVs can become dominant in a nest, and they are part of the core microbiome [4, 33].

### Mixing spiders with different microbiomes

Spiders were collected in November 2019 in Namibia (Table S1), and the microbiome composition of two individual adult female spiders from each nest was analyzed by 16S rRNA gene amplicon sequencing. Three nests with different dominant symbionts were chosen for the experiment. We made this selection based on the most abundant ASVs present in the nests used both for the cross-fostering and mixed microbe experiments, as we know these six ASVs can become dominant in a nest, and they are part of the core microbiome[4, 33]. Randomly chosen individuals from each nest were marked on their abdomen with water-based acrylic paint to track their nest origin. Spiders from nest es16 were mixed with spiders from either nest mm1 or nest es12 into groups of 10 or 12 spiders, and individuals from each nest were represented in three different ratios; 6:6, 2:8, and 8:2, giving two replicates for each ratio. The aim of the experiment was to assess whether and how different proportions of hosts with different dominant symbionts influence homogenization by social transmission. The boxes were examined daily for 39 days, and if any of the individuals had molted, the acrylic paint was re-applied to track the origin of the individuals until the end of the experiment, when all individuals were sampled into 1.5 mL microcentrifuge tubes and stored at −80 °C for DNA extraction and 16S rRNA gene amplicon sequencing. Two spiders died during the experiment.

### DNA extraction, 16S rRNA gene amplicon sequencing, and quantitative PCR

Each spider was homogenized in liquid nitrogen using a microcentrifuge tube pestle directly in the tubes used for collection and storage. After crushing, DNA was extracted using DNeasy Blood and Tissue Kit (Qiagen), following the standard animal tissue protocol. With every round of DNA extraction, a blank (i.e., a tube with no sample) was included as a contamination control.

16S rRNA gene amplicon libraries were prepared according to Illumina’s 16S Metagenomic Sequencing Library Preparation guide, using Bac341F and Bac805R primers [41] to amplify the variable regions V3 and V4. For each round of sequencing, all samples and negative controls were pooled and sequenced on a MiSeq Desktop sequencer (Illumina) using the 2 × 300 bp paired-end sequencing kit according to the provided preparation guide.

qPCR was used to determine the bacterial load in adults and all instars throughout the lifecycle of *S. dumicola*. The bacterial load was determined as the ratio between bacterial 16 S rRNA gene copies and gene copies of a conserved *Stegodyphus* gene as described previously [4].

### 16S rRNA gene amplicon sequencing analysis

All analyses were performed in R v 4.1.2 (R Core Team 2021). Sequences were trimmed to remove barcodes and primers using cutadapt v 0.1.1 [42]. Each sequencing run was processed independently for quality filtering, denoising, and paired-end merging using the R package ‘DADA2’ v. 1.18.0 [43] with filter settings maxEE = (2, 2), truncLen = 230, and truncQ = 2 to identify Amplicon Sequence Variants (ASVs). Data from the different sequencing runs were then merged for chimera finding and classification using DADA2 and Silva SSU reference database nr. 132 [44]. All further data analyses were done using the R packages Phyloseq v. 1.34.0 [45], Microbiome v1.12.0 [46], vegan v2.5.7 [47], ggplot2 v3.3.5 [48], and custom R scripts, if nothing else is specified. After taxonomic classification, the ASVs were filtered to only include those classified as Bacteria. Nucleic acid extraction blanks and PCR negatives were used for decontaminating the data using the R package Decontam v 1.10.0 [49]. Putative contaminants were identified using the prevalence method with a threshold of 0.3 and were subsequently removed from the data.

## Results

### Microbiomes of the *S. dumicola* spiders used in the study

Adult female spiders of all 14 sampled nests had low diversity microbiomes (Shannon index = 0.012 - 1.874), which were strongly dominated (53-100% relative abundance; McNaughton’s dominance index = 0.762-1) by either of the four endosymbionts *Mycoplasma, Diplorickettsia, Ca*. Arachnospira, or *Acaricomes* (Table 1). In addition, some nests consistently harbored a few ASVs of *Staphylococcus* and of an uncultured member of the Weeksellaceae in low abundance.

### Endosymbiont acquisition during spider life cycle

No bacterial 16S rRNA genes were detected in the eggs or first instar, which occurs within the closed egg sac, neither by qPCR nor amplicon PCR assays (Fig. 2). Bacteria were first detected in Instar 2, in which the offspring emerge from the egg sac (Figs. 1, 2). For each of the four nests investigated, all bacterial symbionts (ASVs) carried by the parent generation were already found in the offspring at this stage (Fig. 2). For each nest, also one additional ASV, not found in the adult spiders, was detected in very low abundance and in only some individuals and life stages in the offspring. Overall, the bacterial community composition in the offspring was remarkably similar to that of the adults based on Bray Curtis dissimilarities (Supplementary Fig. S1). We observed a linear relationship between bacterial load (number of 16S rRNA gene copies/number of spider gene copies) and spider body size (Adjusted *R*^2^ = 0.4244, *p* < 0.00001, Supplementary Figure S2). This increase in absolute bacterial abundance varied among ASVs and nests, with *Mycoplasma* load increasing rapidly in all nests and *Ca*. Arachnospira more gradually. In contrast, *Diplorickettsia* load remained low throughout ontogeny and first in the last developmental stages approached the high values seen in the parent generation.

### Effect of cross-fostering on the microbiome of spider offspring

Offspring that remained in their natal nest had a similar microbiome composition to that of the females that reared them (mean Bray-Curtis dissimilarity of 0.316, Wilcoxon rank sum test, FDR-adjusted *p* value < 0.001), which included their biological mothers and female helpers (Fig. 3B, “Control”). Offspring that hatched from egg sacs cross-fostered to a nest with females that carried a different microbiome composition, acquired a microbiome composition similar to that of their foster mothers (mean Bray–Curtis dissimilarity = 0.384, Wilcoxon rank sum test, FDR-adjusted *p*-value < 0.001, Fig. 3B, “Foster mother”), and dissimilar to that of their biological mothers and female helpers (mean Bray–Curtis dissimilarity = 0.724, Fig. 3B, “natal mother”, Wilcoxon rank sum test, FDR-adjusted *p* value < 0.001). Overall, bacterial symbiont composition (at ASV level) of the offspring was always similar to that of the adult female group members with whom they shared the nest (Supplementary Fig. S3).

### Microbiome homogenization upon mixing individuals with different symbionts

**Fig. 4:**
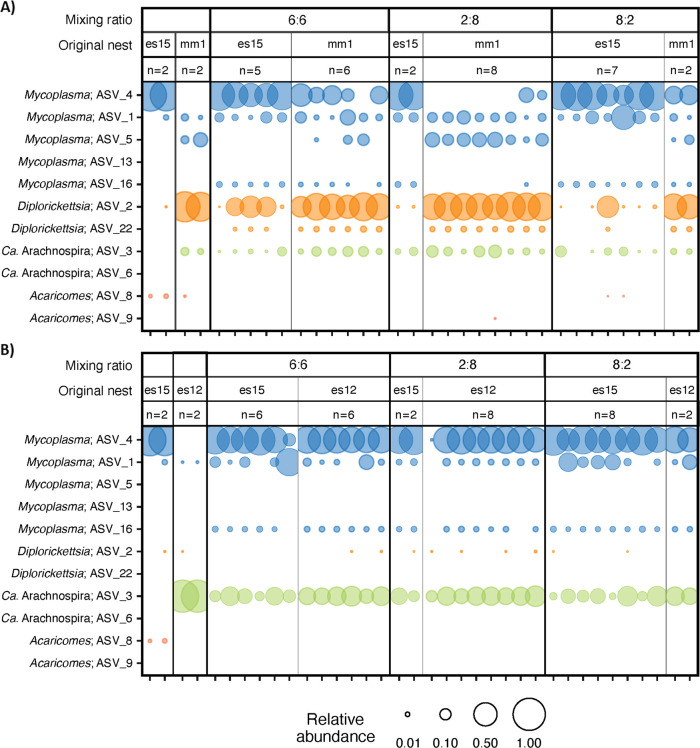
Bubble plot illustrating the relative abundances of symbionts for two mixing experiments. The two left-most panels show the relative abundance of symbionts in the original nests prior to mixing. The remaining panels show the relative abundance of symbionts by the end of the mixing experiment (39 days). The mixing ratios, original nest numbers, and number of individuals are indicated at the top. Bubble size corresponds to the relative abundance according to ASVs in the legend. **A** First mixing experiment. **B** Second mixing experiment.

Spiders were selected from three nests that differed markedly in their microbiomes (Table 1). After combining individuals from two different nests for 39 days in the same box, when an equal number of individuals (six from each nest; 6:6) were combined, the resulting symbiont composition was a combination of the two original microbiomes, and highly similar across all individuals (Fig. 4A, B, Mixing ration 6:6). When combining individuals in unequal ratios (2:8 and 8:2), the transmission of the symbionts was dependant on the type of the symbionts. *Ca*. Arachnospira was transmitted to individuals no matter the ratio and the abundance. *Diplorickettsia* remained in similar abundance in spiders of the original nest but was lower in the newly infected individuals. For *Mycoplasma*, it depended on the ASV type and the context of other ASVs: ASV4 transferred well (also from a few individuals, Fig. 4B) when there was no ASV5 in the mix. But when ASV4 and ASV5 co-occurred (Fig. 4A), they both transferred poorly: ASV5 did not transfer at all, and ASV4 transferred successfully only in the 8:2 ratio. Three ASVs that were not detected in the individuals from the original nests were detected after 39 days of merging individuals: *Diplorickettsia* ASV 22, *Ca*. Arachnospira ASV_9 and *Mycoplasma*_ASV_16. Acaricomes were detected in the original nest but were very rare in the mixed groups.

## Discussion

### Mixed mode transmission maintains endosymbionts between generations in *S. dumicola*

Under the assumption that vertical transmission denotes transmission across generations, i.e., from the parental to the offspring generation, the cross-fostering experiment clearly showed that transmission occurs from the maternal generation (mothers, helping females, or foster mothers) to the offspring, i.e., by vertical transmission. Social living, especially in systems with division of labor and reproductive skew, is expected to exert selection on the symbionts for horizontal transmission [19–21, 28]. We therefore hypothesized that horizontal transmission would also occur in social spiders to avoid a symbiotic dead-end in non-reproducing female helpers [28, 40]. The very low intra-host diversity of the social spider microbiome, on the other hand, with few ASVs dominating each host group ([4, 33]; Figs. 2,4; Table 1), points to a vertical or at least tightly controlled transmission route [50]. The combination of life-cycle analysis and cross-fostering experiments shows that symbionts of the social spider S*. dumicola* are transferred by a combination of horizontal and vertical (but not transovarian) transmission: eggs and instar 1 (both enclosed in the egg sac, Fig. 1) carried no bacterial symbionts; yet as soon as regurgitation feeding started (instar 2, Fig. 1), the offspring acquired microbiomes highly similar to that of the rearing females (Fig. 2). In the cross-fostering experiment, we observed high microbiome similarity between foster mothers and offspring, and low microbiome similarity between cross-fostered offspring and females from their natal nest (Fig. 3). During regurgitation feeding, the tiny offspring are literally attached to the female while feeding on regurgitated fluids from her mouth [39]; it is likely that regurgitated gut content also contains endosymbionts from the gut lumen and gut linings [33]. We did not directly test whether symbionts are transmitted from the biological mother, or from one or more of the helping females. However, the fact that the offspring aquire a microbiome similar to that of the rearing females (whether mother, helper, or foster-mother), combined with the fact that co-occurring females have similar microbiomes, shows high-fidelity transmission across generations by non-transovarian vertical transmission.

A similar mixed-mode of transmission is observed among social and gregarious insects through a combination of mouth-to-mouth feeding (trophallaxis), offspring consumption of the mother’s feces, or regurgitation feeding [21, 51, 52]. In social spiders, regurgitation feeding of offspring occurs in biological mothers as well as in female helpers [38, 39], and our study suggests that regurgitation feeding is both key and sufficient for the vertical symbiont transfer from rearing females to offspring. Although bacterial symbiont transmission via the consumption of infected conspecifics has been observed in isopods [53] and in another (solitary) spider species [54], matriphagy (the consumption of mothers and female helpers by large juveniles) that occurs much later in the spider life cycle (instar 5–7, Fig. 1) is not necessary for reliable symbiont transfer between generations in the social spider *S. dumicola*. Likewise, communal feeding, where spiders share and exchange a mixture of digested prey and digestive liquids [32, 55], only occurs from instar 5 and is not required for transgenerational symbiont transfer.

Mixed mode of transmission of the dominating endosymbionts in *S. dumicola* is consistent with the limited information available for their transmission (or that of their close relatives) in other systems, which all point to tightly controlled but not necessarily transovarian routes: many mycoplasmas are horizontally transmitted either via direct contact or vectors, while in certain subgroups, e.g., the genus *Spiroplasma*, transmission can be vertical or even transovarian [56]. Some *Borrelia* can be transmitted vertically through the ovaries of ticks or insects [57] but most show horizontal transmission via vertebrate hosts [58–60], which may include mixing of several *Borrelia* strains via co-feeding of the arthropod on the same host [61]. The mite pathogen *Acaricomes* [62] is transmitted vertically within a given species but horizontally between different mite and insect species [63]; the transmission mode of the obligate intracellular tick symbiont *Diplorickettsia* is unknown [64]. In conclusion, it seems that the social lifestyle of the spiders has indeed been selected for closely associated endosymbionts. Our study shows that tight host–symbiont associations are maintained by a combination of vertical (but not transovarian) and horizontal transmission routes.

### Social transmission homogenizes microbiome composition within generations in *S. dumicola*

Homogenization of ASV composition between co-inhabiting spiders (Fig. 4) demonstrates that bacterial symbionts are also transmitted socially among adult nest mates. This intragenerational transmission might take place during close contact with adults inside the nest and especially during communal feeding, which, similarly to trophallaxis in insects [21], should facilitate both transmission of symbionts and transfer of nutrients or digestive enzymes. Importantly, intragenerational transmission and homogenization of the individual microbiomes within a nest can occur throughout the life span of *S. dumicola* (Fig. 4), and likely contributes to the long-term stability of the microbiomes observed in *S. dumicola* groups [4]. While environmental sources such as the communal nest theoretically could also act as reservoir to facilitate transmission and homogenization of the spider microbiome, a study of the *S. dumicola* nest microbiome, i.e., the microbes that occupy the actual silk retreat and not the spiders, showed relatively little overlap between the microbiome composition of the spiders and that of their silk nest [65]. This indicates that the silk nest does not mediate environmental transmission of host endosymbionts. In contrast, it is conceivable that *Mycoplasma, Ca*. Arachnospira, *Acaricomes*, or *Diplorickettsia* could be transferred via spider vectors or insect prey (see discussion and references above), potentially leading to mixing with the indigenous microbiome of a spider group. Since the extent of mixing and homogenization appears to be dependent on the mixing ratio (Fig. 4), a singular mixing event will probably not have a lasting imprint on the microbiome of an existing larger group. However, once different symbionts dominate different social groups, repeated transfers or transfers during the founding phase of a new nest may lead to more efficient homogenization and may at least partially explain the divergent microbiomes observed within groups from the same geographic area [4].

In that context, it is interesting to note that, despite the overall high fidelity transmission, not all symbionts and symbiont combinations established equally well: *Mycoplasma* ASV_4 and ASV_5 seem to be almost mutually exclusive (Table 1) and rarely coexisted when mixed together (Fig. 4). This indicates some incompatibility or in-host competition, in agreement with the theoretical prediction that intra-host diversity of symbionts should be low to avoid competition, maybe even host-controlled [50], unless there is a clear functional diversity among the symbionts [66]. Another example is *Acaricomes*, which was poorly established in the mixing experiment (Fig. 4), but was reliably transferred between generations (Fig. 2, SI Table S2), possibly because it requires a certain minimum load in a nest to be infectious [63]. Finally, *Diplorickettsia* was more abundant in the adult life stage than in juveniles, and increased in abundance after the juveniles were participating in communal feeding (from instar 5–7). This increase could be due to access of new resources associated with feed intake and host development. Alternatively, as proposed for the two *Mycoplasma* ASVs above, interactions among bacterial symbionts could play a role in determining the succession and transmission of symbionts across host life stages [67]. For example, changes in abundance of specific symbionts across host life stage were shown in the coral *Orbicella faveolate*, where photosymbionts are beneficial to the adult stage but detrimental to the larval stage [15, 68].Furthermore, competition between symbionts may affect their abundances and functions, as shown for the pea aphid *Acyrthosiphon pisum;* this aphid carries one obligate and several facultative symbionts, *Hamiltonella defensa* and *Rickettsiella viridis*, conferring protection against parasitoid and fungal pathogens respectively. Co-infection of these two symbionts affects the abundance of *H. defensa* and protective functions of *R. viridis* [69]. Moreover, co-infected aphids have reduced survival and fecundity compared to aphids harboring only one symbiont [69, 70]. Whether competition among symbionts or specific trade-offs with life stage applies to the observation that *Diplorickettsia* was more abundant in adult spiders than in juveniles is unknown at this stage, but our results show that *Diplorickettsia* can co-exist with most other symbionts (Table 1, Figs. 2,4).

The genetic background of the host may influence host-microbiome composition, as co-infection or mutual exclusion could be affected by host genetics [71]. Host genetic structure could then generate structure in microbiome composition [72–74]. Despite obligatory inbreeding in our study species *S. dumicola* [32], population genetic analysis showed highly similar genetic composition among nests (groups) within populations due to frequent extinction and colonization events [75]. A separate analysis found no correlation between genetic divergence and microbiome composition of the most prevalent symbionts in *S. dumicola* [33]. It is therefore unlikely that host genetics influences co-infections or mutual exclusions of symbionts in our study system.

### High-fidelity social transmission and sociality

Although horizontal transmission can maintain tight host–symbiont associations, it is expected to be less efficient than vertical transmission in shaping the evolution of obligate associations [10]. Nevertheless, specific conditions such as group living may favor both vertical and horizontal social transmission if tight host-microbe associations are beneficial for the host and/or the symbionts [76, 77]. Horizontal transmission may evolve to enhance transmission fidelity in social animals with reproductive division of labor, to mitigate the risk of vertical transmission of symbionts to non-reproducing workers, which would be a dead-end for the symbiont. The symbiont *Arsenophonus* is transmitted vertically in solitary bees [78], whereas they are horizontally (or both vertically and socially) transmitted in social bees, with higher prevalence of horizontal transmission in non-reproductive workers [29]. This pattern suggests that mixed transmission can evolve in response to the evolution of cooperative breeding. Social transmission facilitiates both modes of transmission and is therefore expected to play a role in shaping stable host–symbiont associations in social systems. In some social insects, the offspring hatch symbiont-free and subsequently acquire bacterial symbionts via close contact with non-reproducing workers (honey bees [25], *Acromyrmex* leaf-cutting ants [18]), emphasizing the role of social transmission in maintaining host–symbiont associations.

In our study of the social spider *S. dumicola*, we found evidence for mixed transmission between generations and social transmission among individuals of the same generation. High temporal stability in microbiome composition between and within generations indicates tight host–symbiont associations in *S. dumicola* [4, 33], highlights the functional significance of social transmission, and suggests relatively high interdependency between the spider host and its symbionts. Whether the mode of group-living shapes microbiome assembly and dynamics, or the microbiome influences various aspects of social behavior, including cooperative breeding [76, 77], remains open research questions.

## Supplementary information

Supplementary Materials

Supplementary table 2

Supplementary table 3

## Data Availability

Sequence data have been submitted to GenBank under BioProject PRJNA830357. ASV sequences, taxonomy, read counts, and relevant metadata can be found in the supplementary material (Tables S2, S3).

## References

[1] Risely A (2020). Applying the core microbiome to understand host–microbe systems. J Anim Ecol.

[2] Denison RF, Kiers ET (2011). Life histories of symbiotic Rhizobia and Mycorrhizal fungi. Curr Biol.

[3] Engel P, Moran NA (2013). The gut microbiota of insects—diversity in structure and function. FEMS Microbiol Rev.

[4] Busck MM, Lund MB, Bird TL, Bechsgaard JS, Bilde T, Schramm A (2022). Temporal and spatial microbiome dynamics across natural populations of the social spider *Stegodyphus dumicola*. FEMS Microbiol Ecol.

[5] Ho P-T, Park E, Hong SG, Kim E-H, Kim K, Jang S-J (2017). Geographical structure of endosymbiotic bacteria hosted by Bathymodiolus mussels at eastern Pacific hydrothermal vents. BMC Evolution Biol.

[6] Breusing C, Genetti M, Russell SL, Corbett-Detig RB, Beinart RA (2022). Horizontal transmission enables flexible associations with locally adapted symbiont strains in deep-sea hydrothermal vent symbioses. Proc Natl Acad Sci.

[7] Sato Y, Wippler J, Wentrup C, Ansorge R, Sadowski M, Gruber-Vodicka H (2022). Fidelity varies in the symbiosis between a gutless marine worm and its microbial consortium. Microbiome..

[8] Bobay L-M, Wissel EF, Raymann K (2020). Strain structure and dynamics revealed by targeted deep sequencing of the honey bee gut microbiome. mSphere..

[9] Hildebrand F, Gossmann TI, Frioux C, Özkurt E, Myers PN, Ferretti P (2021). Dispersal strategies shape persistence and evolution of human gut bacteria. Cell Host Microbe.

[10] Bright M, Bulgheresi S (2010). A complex journey: transmission of microbial symbionts. Nat Rev Microbiol.

[11] Robinson CD, Bohannan BJM, Britton RA (2019). Scales of persistence: transmission and the microbiome. Curr Opin Microbiol.

[12] Ebert D (2013). The epidemiology and evolution of symbionts with mixed-mode transmission. Annu Rev Ecol, Evol Syst.

[13] Russell SL, Pepper-Tunick E, Svedberg J, Byrne A, Ruelas Castillo J, Vollmers C (2020). Horizontal transmission and recombination maintain forever young bacterial symbiont genomes. PLOS Genet.

[14] Bennett GM, Moran NA (2015). Heritable symbiosis: the advantages and perils of an evolutionary rabbit hole. Proc Natl Acad Sci.

[15] Hartmann AC, Marhaver KL, Klueter A, Lovci MT, Closek CJ, Diaz E (2019). Acquisition of obligate mutualist symbionts during the larval stage is not beneficial for a coral host. Mol Ecol.

[16] Kikuchi Y, Hosokawa T, Fukatsu T (2011). Specific developmental window for establishment of an insect-microbe gut symbiosis. Appl Environm Microbiol.

[17] Davidson SK, Stahl DA (2006). Transmission of Nephridial Bacteria of the earthworm *Eisenia fetida*. Appl Environ Microbiol.

[18] Marsh SE, Poulsen M, Pinto-Tomás A, Currie CR (2014). Interaction between workers during a short time window is required for bacterial symbiont transmission in acromyrmex leaf-cutting ants. PLOS One.

[19] Archie EA, Tung J (2015). Social behavior and the microbiome. Curr Opin Behav Sci.

[20] Lombardo MP (2008). Access to mutualistic endosymbiotic microbes: an underappreciated benefit of group living. Behav Ecol Sociobiol.

[21] Onchuru TO, Javier Martinez A, Ingham CS, Kaltenpoth M (2018). Transmission of mutualistic bacteria in social and gregarious insects. Curr Opin Insect Sci.

[22] Sarkar A, Harty S, Johnson KVA, Moeller AH, Archie EA, Schell LD (2020). Microbial transmission in animal social networks and the social microbiome. Nat Ecol Evol.

[23] Raulo A, Allen BE, Troitsky T, Husby A, Firth JA, Coulson T (2021). Social networks strongly predict the gut microbiota of wild mice. ISME J.

[24] Wikberg EC, Christie D, Sicotte P, Ting N (2020). Interactions between social groups of colobus monkeys (Colobus vellerosus) explain similarities in their gut microbiomes. Anim Behav.

[25] Martinson VG, Moy J, Moran NA (2012). Establishment of characteristic gut bacteria during development of the honeybee worker. Appl Environ Microbiol.

[26] Koch H, Abrol DP, Li J, Schmid-Hempel P (2013). Diversity and evolutionary patterns of bacterial gut associates of corbiculate bees. Mol Ecol.

[27] Aanen DK, Eggleton P, Rouland-Lefèvre C, Guldberg-Frøslev T, Rosendahl S, Boomsma JJ (2002). The evolution of fungus-growing termites and their mutualistic fungal symbionts. Proc Natl Acad Sci.

[28] Frost CL, Pollock SW, Smith JE, Hughes WOH (2014). Wolbachia in the flesh: symbiont intensities in germ-line and somatic tissues challenge the conventional view of wolbachia transmission routes. PLOS One.

[29] Drew GC, Budge GE, Frost CL, Neumann P, Siozios S, Yañez O (2021). Transitions in symbiosis: evidence for environmental acquisition and social transmission within a clade of heritable symbionts. ISME J.

[30] Poulsen M, Bot ANM, Currie CR, Nielsen MG, Boomsma JJ (2003). Within-colony transmission and the cost of a mutualistic bacterium in the leaf-cutting ant Acromyrmex octospinosus. Funct Ecol.

[31] Kwong WK, Moran NA (2016). Gut microbial communities of social bees. Nat Rev Microbiol.

[32] Lubin Y, Bilde T. The evolution of sociality in spiders. advances in the study of behavior. Academic Press; 2007. 37. 83–145.

[33] Busck MM, Settepani V, Bechsgaard J, Lund MB, Bilde T, Schramm A (2020). Microbiomes and specific symbionts of social spiders: compositional patterns in host species, populations, and nests. Front Microbiol.

[34] Vanthournout B, Busck MM, Bechsgaard J, Hendrickx F, Schramm A, Bilde T (2018). Male spiders control offspring sex ratio through greater production of female-determining sperm. Proc R Soc B: Biol Sci.

[35] Keiser CN, Hammer TJ, Pruitt JN (2019). Social spider webs harbour largely consistent bacterial communities across broad spatial scales. Biol Lett..

[36] Keiser CN, Pinter-Wollman N, Augustine DA, Ziemba MJ, Hao L, Lawrence JG (2016). Individual differences in boldness influence patterns of social interactions and the transmission of cuticular bacteria among group-mates. Proc R Soc B: Biol Sci.

[37] Walter A, Bilde T (2022). Avoiding the tragedy of the commons: Improved group-feeding performance in kin groups maintains foraging cooperation in subsocial Stegodyphus africanus spiders (Araneae, Eresidae). J Evol Biol.

[38] Junghanns A, Holm C, Schou MF, Overgaard J, Malte H, Uhl G, et al. Physiological adaptations to extreme maternal and allomaternal care in spiders. Front Ecol Evol. 2019;7.

[39] Junghanns A, Holm C, Schou MF, Sørensen AB, Uhl G, Bilde T (2017). Extreme allomaternal care and unequal task participation by unmated females in a cooperatively breeding spider. Anim Behav.

[40] Van Borm S, Wenseleers T, Billen J, Boomsma JJ (2001). Wolbachia in leafcutter ants: a widespread symbiont that may induce male killing or incompatible matings. J Evol Biol.

[41] Herlemann DPR, Labrenz M, Jürgens K, Bertilsson S, Waniek JJ, Andersson AF (2011). Transitions in bacterial communities along the 2000 km salinity gradient of the Baltic Sea. The. ISME J.

[42] Martin M (2011). Cutadapt removes adapter sequences from high-throughput sequencing reads. EMBnet J.

[43] Callahan BJ, McMurdie PJ, Rosen MJ, Han AW, Johnson AJA, Holmes SP (2016). DADA2: High-resolution sample inference from Illumina amplicon data. Nat Methods.

[44] Quast C, Pruesse E, Yilmaz P, Gerken J, Schweer T, Yarza P (2012). The SILVA ribosomal RNA gene database project: improved data processing and web-based tools. Nucleic Acids Res.

[45] McMurdie PJ, Holmes S (2013). phyloseq: an R package for reproducible interactive analysis and graphics of microbiome census data. PLOS One.

[46] Leo Lahti. SS Tools for microbiome analysis in R. 2012–2019.

[47] Oksanen J, Blanchet, FG, Kindt, R, Legendre, P, Minchin, PR, O’Hara, RB, et al. vegan: Community Ecology Package. 2012.

[48] Wickham H. ggplot2: Elegant Graphics for Data Analysis: Springer-Verlag New York; 2016.

[49] Davis NM, Proctor DM, Holmes SP, Relman DA, Callahan BJ (2018). Simple statistical identification and removal of contaminant sequences in marker-gene and metagenomics data. Microbiome..

[50] Frank SA (1996). Host–symbiont conflict over the mixing of symbiotic lineages. Proc R Soc Lond Ser B: Biol Sci.

[51] Scott MP, Traniello JFA (1990). Behavioural and ecological correlates of male and female parental care and reproductive success in burying beetles (Nicrophorus spp.). Anim Behav.

[52] Shukla SP, Vogel H, Heckel DG, Vilcinskas A, Kaltenpoth M (2018). Burying beetles regulate the microbiome of carcasses and use it to transmit a core microbiota to their offspring. Mol Ecol.

[53] Le Clec’h W, Chevalier FD, Genty L, Bertaux J, Bouchon D, Sicard M (2013). Cannibalism and predation as paths for horizontal passage of wolbachia between terrestrial isopods. PLOS One.

[54] Su Q, Hu G, Yun Y, Peng Y (2019). Horizontal transmission of Wolbachia in Hylyphantes graminicola is more likely via intraspecies than interspecies transfer. Symbiosis..

[55] Walter A, Bechsgaard J, Scavenius C, Dyrlund TS, Sanggaard KW, Enghild JJ (2017). Characterisation of protein families in spider digestive fluids and their role in extra-oral digestion. BMC Genom.

[56] Razin S. The genus mycoplasma and related genera (Class Mollicutes). In: Dworkin M, Falkow S, Rosenberg E, Schleifer K-H, Stackebrandt E, editors. The Prokaryotes: Volume 4: Bacteria: Firmicutes, Cyanobacteria. New York, NY: Springer US; 2006. 836–904.

[57] Rollend L, Fish D, Childs JE (2013). Transovarial transmission of *Borrelia spirochetes* by *Ixodes scapularis*: A summary of the literature and recent observations. Ticks Tick-borne Dis..

[58] Han S, Lubelczyk C, Hickling GJ, Belperron AA, Bockenstedt LK, Tsao JI (2019). Vertical transmission rates of *Borrelia miyamotoi* in *Ixodes scapularis* collected from white-tailed deer. Ticks and Tick-borne. Diseases..

[59] Melaun C, Zotzmann S, Santaella VG, Werblow A, Zumkowski-Xylander H, Kraiczy P (2016). Occurrence of *Borrelia burgdorferi* s.l. in different genera of mosquitoes (Culicidae) in Central Europe. Ticks Tick-borne Dis..

[60] Barbour AG, Hayes SF (1986). Biology of Borrelia species. Microbiol Rev.

[61] States SL, Huang CI, Davis S, Tufts DM, Diuk-Wasser MA (2017). Co-feeding transmission facilitates strain coexistence in *Borrelia burgdorferi*, the Lyme disease agent. Epidemics.

[62] Schütte C, Gols R, Kleespies RG, Poitevin O, Dicke M (2008). Novel bacterial pathogen *Acaricomes phytoseiuli* causes severe disease symptoms and histopathological changes in the predatory mite *Phytoseiulus persimilis* (Acari, Phytoseiidae). J Invertebr Pathol.

[63] Xie Z, Hoffmann AA, Zhang B, Xu X (2022). Detection, detrimental effects, and transmission pathways of the pathogenic bacterium acaricomes phytoseiuli in commercial predatory mites. microbiology. Spectrum..

[64] Mediannikov O, Sekeyová Z, Birg M-L, Raoult D (2010). A novel obligate intracellular gamma-proteobacterium associated with ixodid ticks, *Diplorickettsia massiliensis*, Gen. Nov., Sp. Nov. PLOS One.

[65] Nazipi S, Elberg CL, Busck MM, Lund MB, Bilde T, Schramm A (2021). The bacterial and fungal nest microbiomes in populations of the social spider *Stegodyphus dumicola*. Syst Appl Microbiol.

[66] Ansorge R, Romano S, Sayavedra L, Porras MÁG, Kupczok A, Tegetmeyer HE (2019). Functional diversity enables multiple symbiont strains to coexist in deep-sea mussels. Nat Microbiology.

[67] Ferrari J, Vavre F (2011). Bacterial symbionts in insects or the story of communities affecting communities. Philos Trans R Soc B: Biol Sci.

[68] Hartmann AC, Baird AH, Knowlton N, Huang D (2017). The paradox of environmental symbiont acquisition in obligate mutualisms. Current Biology.

[69] Leclair M, Polin S, Jousseaume T, Simon J-C, Sugio A, Morlière S (2017). Consequences of coinfection with protective symbionts on the host phenotype and symbiont titres in the pea aphid system. Insect Sci.

[70] Oliver KM, Moran NA, Hunter MS (2006). Costs and benefits of a superinfection of facultative symbionts in aphids. Proc R Soc B: Biol Sci.

[71] Chaston JM, Dobson AJ, Newell PD, Douglas AE (2016). Host genetic control of the microbiota mediates the drosophila nutritional phenotype. Appl Environm Microbiol.

[72] Yuan ML, Dean SH, Longo AV, Rothermel BB, Tuberville TD, Zamudio KR (2015). Kinship, inbreeding and fine-scale spatial structure influence gut microbiota in a hindgut-fermenting tortoise. Mol Ecol.

[73] Brinker, P., Chen, F., Chehida, Y. B., Beukeboom, L. W., Fontaine, M. C. & Salles, J. F. Microbiome composition is shaped by geography and population structure in the parasitic wasp Asobara japonica, but not in the presence of the endosymbiont Wolbachia. Mol Ecol. 2022;1–15. 10.1111/mec.16699. 10.1111/mec.16699 36125236

[74] Easson CG, Chaves-Fonnegra A, Thacker RW, Lopez JV (2020). Host population genetics and biogeography structure the microbiome of the sponge *Cliona delitrix*. Ecol Evol.

[75] Settepani V, Bechsgaard J, Bilde T (2016). Phylogenetic analysis suggests that sociality is associated with reduced effectiveness of selection. Ecol Evol.

[76] Cusick JA, Wellman CL, Demas GE (2021). The call of the wild: using non-model systems to investigate microbiome–behaviour relationships. J Exp Biol.

[77] Lewin-Epstein O, Aharonov R, Hadany L (2017). Microbes can help explain the evolution of host altruism. Nat Commun.

[78] Nováková E, Hypša V, Moran NA (2009). Arsenophonus, an emerging clade of intracellular symbionts with a broad host distribution. BMC Microbiol.

