# Supplementary material for: Social transmission of bacterial symbionts homogenizes the microbiome within and across generations of group-living spiders
Source: ISME Commun. 2023 Jun 17;3:60. doi: 10.1038/s43705-023-00256-2 (PMC10276852; doi:10.1038/s43705-023-00256-2)
Supplement: Supplementary file 1 — Supplementary Materials [file 43705_2023_256_MOESM1_ESM.docx]

Supplementary information for

**Social transmission of bacterial symbionts homogenizes the microbiome within and across generations of group-living spiders**

Clémence Rose, Marie B. Lund, Andrea M. Søgård, Mette M. Busck, Jesper S. Bechsgaard, Andreas Schramm, Trine Bilde

Table S1: This document. List of nests

Table S2: excel file. ASV table with sequences, taxonomy, and read counts

Table S3: excel file. Metadata for all samples

Figure S1: This document. Life cycle experiment. Bray-Curtis dissimilarities between mothers and individual instars

Figure S2: This document. Life cycle experiment. Bacterial load as a function of spider size

Figure S3: This document. Cross-fostering experiment: Heatmap of most abundant symbionts in all spiders from the two cross-fostering experiments

| **Table S1.** List of nests used in the three different experiments | | | | | |
| --- | --- | --- | --- | --- | --- |
|  | **Nest ID** | **Collection site** | **Lat** | **Long** | **Collection date** |
| ***Life Cycle*** | | | | | |
|  | lc1 | Otavi, Namibia | -18.78351 | 17.27393 | Spring 2017 |
|  | lc2 | Otavi, Namibia | -19.47742 | 17.19411 | 17/Jan/2019 |
|  | lc3 | Otavi, Namibia | -19.47703 | 17.19427 | 17/Jan/2019 |
|  | lc4 | Otavi, Namibia | -19.47892 | 17.19469 | 18/Jan/2019 |
| ***Cross fostering*** | | | | | |
|  | es1 | Central district, Botswana | -22.889199 | 26.944915 | 20/Oct/2018 |
|  | es2 | Central district, Botswana | -22.889199 | 26.944921 | 25/Oct/2018 |
|  | es3 | Central district, Botswana | -22.723295 | 27.048710 | 25/Oct/2018 |
|  | es4 | Central district, Botswana | -22.864167 | 26.9598060 | 25/Oct/2018 |
|  | es5 | Central district, Botswana | -22.891009 | 26.944046 | 25/Oct/2018 |
|  | es6 | Central district, Botswana | -22.676596 | 27.073735 | 25/Oct/2018 |
|  | es10 | Mahalapye, Botswana |  |  |  |
|  | es11 | Khomas Region, Namibia | -22.57342 | 17.22004 | 19/Nov/2019 |
|  | es12 | Otjozondjupa Region, Namibia | -19.09596 | 17.22699 | 19/Nov/2019 |
|  | es13 | Omaheke region, Namibia | -21.16688 | 18.27093 | 17/Nov/2019 |
|  | es14 | Omaheke region, Namibia | -21.16708 | 18.27068 | 17/Nov/2019 |
|  | es15 | Otjozondjupa Region, Namibia | -21.16643 | 18.27049 | 17/Nov/2019 |
|  | es16 | Palapye, Botswana | -22.96658 | 26.89183 | 09/Nov/2019 |
| ***Mixed microbe*** | | | | | |
|  | mm1 | Mahalapye, Botswana | -22.96658 | 26.89183 | 09/Nov/2019 |
|  | es15 | Otjozondjupa Region, Namibia | -21.16643 | 18.27049 | 17/Nov/2019 |
|  | es12 | Oshikoto Region, Namibia | -19.09596 | 17.22699 | 19/Nov/2019 |

**Figure S1.**

Life cycle experiment. Bray-Curtis dissimilarity between adults and each of the Instars after emergence from the egg sac. Colors correspond to the four different nests.


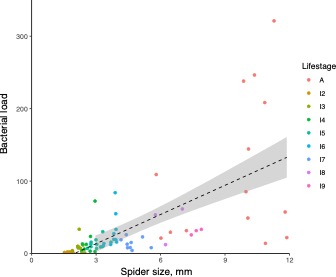


**Figure S2.**

Life cycle experiment. Bacterial load (number of 16S rRNA gene copies/number of spider gene copies) as a function of spider size. Colors show the different life stages: A = adult, I2 = instar 2. etc. The dashed line is linear regression with 95% confidence interval.

Adjusted R^2^= 0.4244, p <0.00001

**Figure S3**. *Continues next page*

**Figure S3.** *Continued from previous page*. Heatmap of the most abundant symbionts in the cross-fostering experiment. Colour scale shows the relative abundance of each ASV. Top facet shows the foster nest, where the spiderlings were raised. Second facet shows the experimental boxes. Third facet shows mothers or spiderlings. Fourth facet shows the natal nest. Panel A-C is data from the second cross fostering experiment and panel D and E is from the first cross fostering experiment.
